# The relationship between abuse and neglect and adolescent suicidality: A moderated mediation model

**DOI:** 10.3389/fpsyg.2022.1019878

**Published:** 2022-11-17

**Authors:** Hongjuan Chang, Zhenzhu Yao, Yu Zhang, Jiaqi Chen, Peipei Shi

**Affiliations:** ^1^School of Nursing, Xinxiang Medical College, Xinxiang, China; ^2^Graduate School of China Medical University, Shenyang, China; ^3^School of Nursing and Health, Henan University, Kaifeng, China; ^4^College of Life Science and Technology, Xinxiang Medical College, Xinxiang, China

**Keywords:** suicidality, resilience, moderated mediation, adolescents, rural China

## Abstract

Abuse and neglect can lead to suicide in adolescents; however, the internal mechanisms between abuse and neglect and suicidality remain unclear. We hypothesized that abuse and neglect could affect adolescent suicidality, and that mediating and moderating mechanisms exist between these two variables. We tested the mediating effects of stressful life events (misunderstanding and discrimination by others, poor academic performance, failed exams, interpersonal problems, and school punishment) on suicidal tendencies and moderating effects of resilience among 5,852 adolescents from 37 middle schools in five provinces of North, south, East, west, and central China. We used a questionnaire to collect data and adopted structural equation modeling to verify the correlation, mediating, and moderating effects among the variables. The results showed that stressful life events mediated the relationship between abuse, neglect, and suicide. Furthermore, resilience moderated the direct effect and second half of the mediating effect.

## Introduction

Suicide is currently the second leading cause of death in adolescents, according to global statistics. Approximately 7% of the youth reported one or more suicide attempts in the past year (Kann et al., 2018). Recent estimates from the Centers for Disease Control and Prevention’s 2019 Youth Risk Behavior Survey suggested that approximately 19% of high school students had suicidal thoughts in the past year, and 9% attempted suicide at least once (Ivey-Stephenson et al., 2020). Strikingly, China’s suicide rate among adolescents was among the highest in the world, with the prevalence of suicidality ranging from 2.7 to 45.1% (Lew et al., 2020). As suicide is a potentially preventable public health issue, it is important to study the causes of suicidality to improve future public health interventions (Sampasa-Kanyinga et al., 2015). In recent years, the influence of abuse and neglect (Stickley et al., 2020), and stressful life events (Brailovskaia et al., 2020) on individual suicidality has gradually attracted the attention of researchers and society.

### Abuse, neglect and suicide

Abuse and neglect are a serious public health problem worldwide. Abuse is an act comprising all forms of physical and/or emotional abuse, sexual abuse, neglect, commercial exploitation, or other exploitation of the child, causing actual or potential harm to the health, survival, development, or dignity of the child in the context of a relationship of responsibility, trust, or power. Neglect refers to the primary caregiver’s failure to provide for a child’s necessary physical, psychological, or educational needs, or the failure to protect the child from harm (and potential harm; Stoltenborgh et al., 2015). Several studies have confirmed that children worldwide suffer from high levels of abuse and neglect in childhood (Kim et al., 2017; Wen et al., 2021). Asian children suffer from great abuse and neglect; the total incidence is as high as 75.63%. The incidences of physical, emotional, and sexual abuse and physical and emotional neglect were 45.65, 20.05, 12.46, 10.26, and 28.34%, respectively (Pace et al., 2022). Child abuse and neglect are also prevalent in China, with incidence of physical abuse, emotional abuse, sexual abuse, and neglect in children under 18 years of age at 26.6, 19.6, 8.7, and 26.0%, respectively (Fang et al., 2015). Abuse and neglect can negatively affect the physical and mental health as well as behavioral functioning of individuals. Furthermore, it can threaten children’s physical, mental, and emotional development (Belvederi Murri et al., 2018).

Previous studies have shown that abuse and neglect play an important role in the occurrence and development of suicidal thoughts and are closely associated with individual suicides (Zatti et al., 2017). First, according to the interpersonal theory of suicide, childhood abuse and neglect promote children’s adaptation to the painful state and reduce their fear of death, thereby gradually advancing the individual’s ability to commit suicide. Furthermore, the schematic evaluation model of suicide highlights emphasizes that the poor information processing bias will affect the individual evaluation system and help form the suicide schema. Childhood abuse and neglect can gradually worsen the individual’s perception of failure and pain, destroy the individual’s evaluation of stress and pressure, and make them tend to choose the direct means to escape failure and pain -- suicide (Williams et al., 2005; Johnson et al., 2008). The results of the empirical study also found that abuse and neglect positively correlated with suicidal behaviors (Palmer et al., 2021). Therefore, we hypothesized that abuse and neglect positively predict suicidal behavior (H1).

### The mediating effect of stressful life events

Adolescence is an important transition period and a vulnerable developmental stage for individual learning; cognition; and social, physiological, and physical changes, which are affected by stressful life events (Byrne et al., 2007). Stressful life events experienced by individuals in the different age groups are different. The negative life events experienced by adolescents mainly include misunderstanding and discrimination by others, neglect and/or abuse by families, poor academic performance, failure in exams, problems in romantic relationships, and punishment at school (Brailovskaia et al., 2020).

Researchers have clearly established a direct link between childhood abuse, neglect, and suicide risk. This association’s internal mechanisms have received much attention from scholars. However, there are gaps in the literature regarding the underlying mechanisms of the relationship between abuse, neglect, and suicide, especially in adolescent populations (Porter et al., 2020). Based on Han kin’s stress generation theory and Ham men’s stress sensitivity hypothesis, as well as physiological analysis, childhood abuse and neglect can positively predict the stressful life events experienced by individuals. First, according to stress generation theory, childhood abuse and neglect as chronic stressors can trigger additional stressors later in life, causing individuals to experience more stressful life events (Hankin, 2005). According to the stress sensitivity hypothesis, the chronic stress experienced in early childhood exacerbates the acquisition of recent life stress (Hammen et al., 2000). In other words, adolescents facing stressful life events are more negatively affected by stressful events if they are abused and neglected during childhood. From a physiological perspective, chronic stressors in childhood (abuse and neglect) can disrupt physiological systems that process an individual’s response to stress, such as the hypothalamic–pituitary–adrenal (HPA) axis and autonomic nervous system (Loman and Gunnar, 2010; Alink et al., 2012; McLaughlin et al., 2015). Adolescents who have experienced child abuse cannot cope with stressful events without developing physiological responses to adapt to stress. However, previous studies have confirmed that stressful life events have always been an important predictive factor for suicide in adolescents (Duprey et al., 2021). Stressful life events are significantly associated with individual suicidal behaviors, suicide attempts, and suicidal ideation (Lee et al., 2018), and a positive dose–response relationship with suicide risk (Serafini et al., 2015). In addition, empirical studies have confirmed that adolescents suffering from childhood abuse and neglect are more likely to develop psychological disorders and traumatic stress in the face of stressful life events, which may lead to suicide among adolescents (Duprey et al., 2021). Therefore, we hypothesized that childhood abuse and neglect may have an indirect effect on individual suicidal behaviors through the mediating effect of stressful life events (H2).

### Resilience as a moderator

In contrast to risk factors, the influence of protective factors on adolescent suicidality is not clear (Zhu et al., 2019). To date, research on potential mechanisms for protection factors on adolescents’ suicide has been carried out. Our research has advocated the role of resilience in suicide, not only enriching research in data information, but also providing a new direction and target intervention to resolve this issue.

Resilience is an ability, concept, or set of beliefs that buffer an individual against the negative effects of risk factors or stressors (Wu et al., 2021). However, not all individuals who experience chronic (childhood abuse and neglect) and acute (stressful life events) stressors engage in suicidal behavior. This may be due to the presence of protective factors (resilience) that moderate the effects of chronic and acute stressors on suicide. First, according to the adjustment model of resilience, resilience can prevent the occurrence of suicidal behaviors by playing a regulatory role in the development of risk factors for suicidal outcomes (Siegmann et al., 2018). In other words, risk factors (childhood abuse and stressful life events) are associated with a higher risk of suicide; however, resilience can weaken this association, thereby reducing or eliminating the risk of suicide caused by risk factors. Furthermore, the protective model of resilience underlines that mutual restriction of protective and risk factors can help avoid harmful consequences. Protective factors can not only weaken the harm of risk factors to individuals but also reconcile the relationship between risk and adverse consequences (Garmezy, 1985). In other words, childhood abuse and stressful life events as risk factors interact with resilience to protect individuals from suicidal behavior. In the face of childhood abuse and stressful life events, a high resilience levels can promote individual adaptation, help individuals overcome adversity and setbacks, and promote growth (Angel, 2016). Therefore, we hypothesized that resilience may play a moderating role in the relationship between child abuse and neglect and suicide, stressful life events, and suicidal behaviors; that is, the direct predictive effect of childhood abuse and neglect on suicidal behavior and the mediating effect of stressful life events are both affected by resilience (H3).

**Figure 1:**
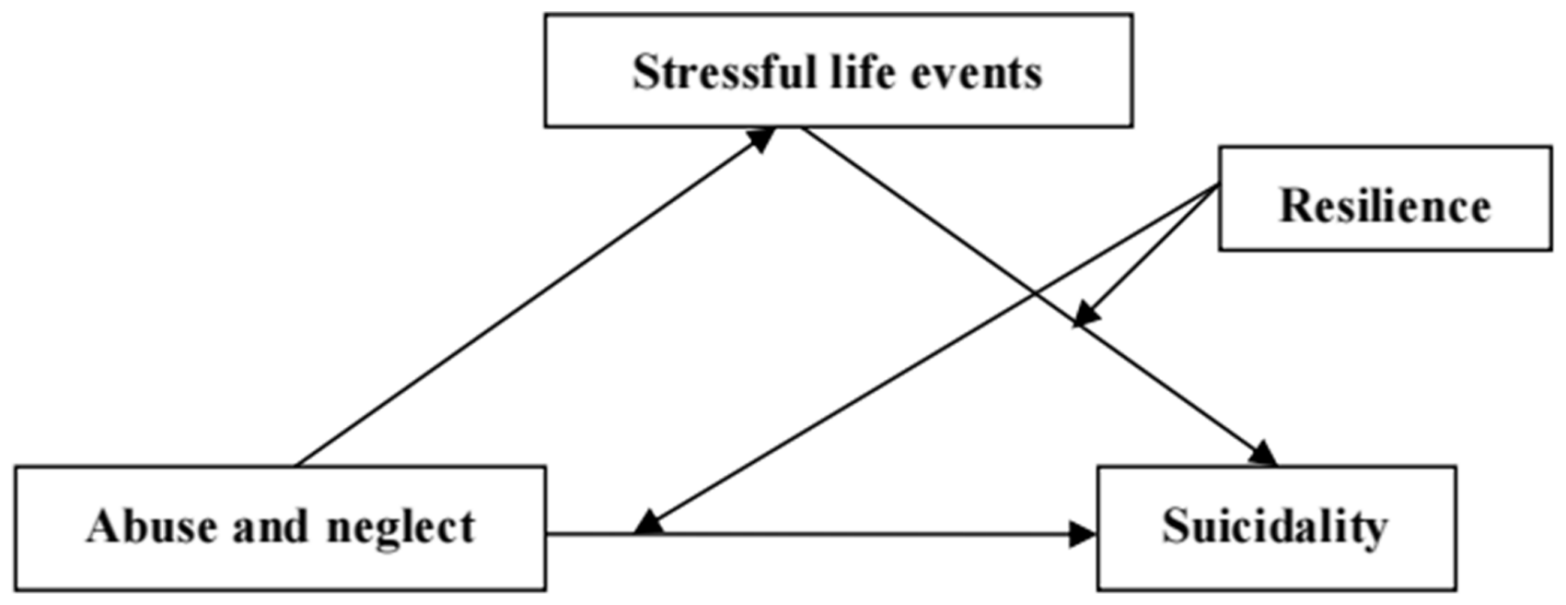
Hypothesized model illustrating moderated mediation.

Considering the high incidence of abuse and neglect in childhood and the grave consequences of suicide, it is imperative to study the underlying mechanisms of the link between abuse and neglect and suicidal behavior. Given that stressful life events link abuse and neglect and suicidal behavior (Marques-Feixa et al., 2021), this study examined the mediating role of stressful life events between the aforementioned behaviors. Furthermore, resilience, as a protective factor, attenuates the impact of risk factors on individual adverse behaviors (Yu et al., 2021). Therefore, we used resilience as a moderator to examine the strength of the direct relationship between abuse and neglect and adolescent suicidal behavior. The proposed model is illustrated in Figure 1.

## Materials and methods

### Participants

The data were part of a nationwide study on mental health outcomes among adolescents in rural China, which was initiated in 2022. We adopted a multistage cluster random sampling method to collect data to represent all students in rural China. In the first stage, five provinces were selected from the north, south, east, west, and middle parts of the country on behalf of the entire rural China. In the second stage, one county was randomly chosen from each province. In the final stage, schools were selected based on their reported enrollment sizes. Inclusion criteria: (1) middle school students aged 10–18; (2) Lucid, good communication and understanding skills; (3) Informed consent and voluntary participation. Exclusion criteria: mental disorders or other serious diseases.

In total, 6,189 students were recruited, and a consent letter was forwarded to their parents or guardians. Among the responders, 337 were excluded because they submitted incomplete questionnaires. In total, 5,852 students (3,042 boys and 2,810 girls) were included in our analysis. The participants’ age ranged from 12 to 16 years with a mean age of 14.01 (*SD* = 0.79). None of them was diagnosed with any other condition, as shown in Table 1. The response rate of participants was 94.55% (5,852/6,189). We obtained informed consent from the parents or guardians of all participants. Data was collected by a group of participants in the school-unified training of psychological teachers, who explained the purpose and procedures of the study to participants. The students were instructed to complete the questionnaire anonymously and to respond to all questions honestly. We advised participants to place completed questionnaires in envelopes and not to hand them directly to schoolteachers or school personnel. They were told that the data would be used for scientific research only and confidentiality would be retained. Informed consent was obtained from the school, parents, and students, each of whom signed the informed consent form. The ethical protocol, including the questionnaires, was approved by the targeted schools and ethics committee of Xinxiang Medical College (XYLL-2018015). The study was conducted according to approved guidelines.

**Table 1:** Demographic characteristics and related variables of study participants (*n* = 5,852).

Measure		Mean ± SD/*n* (%)
Gender		
	Boy	3,042 (52.0)
	Girl	2,810 (48.0)
Age		14.01 ± 0.79
Grade		
	Seven	1,602 (27.4)
	Eight	2,172 (37.1)
	Nine	1,632 (27.9)
	Ten	420 (7.2)
	Eleven	26 (0.4)
Province		
	Zhejiang	1,584 (27.1)
	Guizhou	852 (14.6)
	Henan	1,085 (18.5)
	Anhui	1,420 (24.3)
	Heilongjiang	911 (15.6)
School type		
	Public school	5,406 (92.4)
	Private school	446 (7.6)
Social and economic status		
	≥9,000	344 (5.9)
	7,000 ~ 8,999	541 (9.2)
	5,000 ~ 6,999	1,167(19.9)
	3,000 ~ 4,999	2,116 (36.2)
	2000 ~ 2,999	804 (13.7)
	<2000	880 (15.1)

### Measurement of structures

#### Neglect and abuse

We used a validated Chinese version of the Parents-Child Conflict Tactics Scale (CTSPC) to measure the prevalence of neglect and abuse. This scale is commonly used in China to assess subjective feelings of neglect and physical abuse in children or adolescents (Straus et al., 1998). The scale consists of 17 items with four factors: neglect, corporal punishment, physical maltreatment, and severe physical maltreatment. The participants in our study were requested to rate how frequently they encountered the listed behaviors in the past year using a three-point Likert scale: covering none (1), once (2), twice, or more (3). Neglect and abuse scores were calculated by summing up the frequency of behaviors in all groups. In the present study, Cronbach’s α was 0.81.

#### Stressful life events

The Adolescent Self-Rating Life Events Check List (ASRLEC), a 5-point Likert scale, assesses the participants’ life events as well as the consequent effects, if any, in the past 12 months (Peng et al., 2012). The scale comprises 27 items with six factors: interpersonal relationships, study pressure, being punished, bereavement, change for adaptation, and others. Responses were rated from 1 (not at all) to 5 (very much); the higher the score, the greater the life pressure. This scale is generally used to measure stress levels among Chinese students. In the present study, Cronbach’s α was 0.92.

#### Psychological resilience scale

The Chinese Resilience Scale is a 27-item, 5-point Likert-type assessment that measures the ability to cope with stress and adversity on five factors: goal planning, help seeking, family support, affect control, and positive thinking (Jung et al., 2012). Respondents rated the items from 1 (totally inconsistent with) to 5 (in full compliance with), with higher scores reflecting greater resilience. The Chinese version of the Resilience Scale has good reliability and validity in Chinese children. In the present study, Cronbach’s α was 0.79.

#### Suicidality

We used the World Health Organization (WHO) Composite International Diagnostic Interview (CIDI) to assess suicidality (Robins et al., 1988). Suicidal ideation (“Have you ever seriously thought about committing suicide?”), plans (“Have you ever made a plan for committing suicide?”), gestures (“Have you ever prepared gestures to attempt suicide?”), and attempts (“Have you ever attempted suicide?”) were assessed. Item responses were rated on a 3-point scale ranging from 0 to 2 and the final score was computed. In the present study, Cronbach’s α was 0.80.

### Data analyses

Individual responses with missing values were deleted to ensure the quality and reliability of the data. We z-transformed all variables before entering them into the model to compare the effect sizes and reduce multicollinearity (Allan and Duffy, 2012). Sex and age were included as covariates in the analyses. We used SPSS macro-PROCESS developed by Preacher and Hayes to conduct moderated mediation analysis.^1^ First, a regression model controlling for stressful life events was developed to examine the effects of abuse and neglect on suicide. Next, bivariate Pearson correlations were conducted to examine basic associations among the variables, and demographic variables were controlled as covariates in the subsequent models. We conducted moderated mediation analyses based on 5,000 bootstrapped samples using Models 1 and 15 and accelerated the 95% confidence intervals (CIs). A *p* < 0.05 was considered statistically significant in correlation analyses. The indirect effect of 95% CIs not including zero was statistically significant at the 0.05 level (Preacher and Hayes, 2008; Hayes, 2013). Conventional procedures were applied to plot simple slopes to interpret interactive effects.

## Results

### Descriptive statistics and correlation analysis

The results of the descriptive statistics and correlation analyses are presented in Table 2. We found that abuse and neglect positively correlated with suicidality and stressful life events. Furthermore, a positive correlation between stressful life events and suicidality was established.

**Table 2:** Descriptive statistics and correlations (*N* = 5,852).

Variables	1	2	3	4	5	6	M	SD
1.Age	−						14.01	0.792
2.Gender	−0.014	−					1.48	0.500
3.Stressful life events	0.039**	−0.046**	−				53.49	19.004
4.Abuse and neglect	−0.015	−0.048**	0.404**	−			4.51	4.806
5.Resilience	0.035**	0.086**	−0.295**	−0.285**	−		91.65	13.283
6.Suicidality	0.000	0.036**	0.294**	0.287**	−0.238**	−	0.41	1.227

*p < 0.05; **p < 0.01.

### The impact of abuse and neglect on suicide

The effects of abuse and neglect on suicide are shown in Table 3. After controlling for negative life events, abuse and neglect can directly and positively predict adolescent suicide.

**Table 3:** The impact of abuse and neglect on suicidality.

	*B*	*SE*	*t*	*P*
(constant)	0.080	0.021	3.786	<0.001
Abuse and neglect	0.073	0.003	22.958	<0.001

***p < 0.001, **p < 0.01, *p < 0.05.

### Testing for mediating effect

The mediating effects of stressful life events are shown in Table 4. The total effect of abuse and neglect on suicidality in the absence of stressful life events was significant (*β* = 0.29, *t* = 23.16, and *p* < 0.001). Additionally, abuse and neglect had a positive effect on stressful life events (*β* = 0.40, *t* = 33.78, and *p* < 0.001). When stressful life events were added to the analysis as mediators, the effects of abuse and neglect remained significant (*β* = 0.20, *t* = 15.16, and *p* < 0.001), and stressful life events remained predictive of suicidality (*β* = 0.21, *t* = 15.99, and *p* < 0.001). Bootstrapping indicated that there was a significant role of stressful life events in explaining the association between abuse and neglect, and suicidality (indirect effect = 0.09, *95%CI* = 0.07–0.10); as shown in Table 5.

**Table 4:** Mediation model testing of stressful life events.

IV	Model 1 (DV: Suicidality)		Model 2 (DV: Stressful life events)		Model 3 (DV: Suicidality)	
	*β*	*t*	*β*	*t*	*β*	*t*
Gender	0.050	4.025***	−0.026	−2.137*	0.056	4.557***
Age	0.005	0.373	0.044	3.717***	−0.005	−0.397
Abuse and neglect	0.290	23.155***	0.404	33.776***	0.203	15.176***
Stressful life events					0.215	15.994***
*R^2^*	0.085		0.166		0.124	
*F*	181.552***		338.537***		206.051***	

***p < 0.001, **p < 0.01, *p < 0.05. DV, dependent variable; IV, independent variable.

**Table 5:** Mediating effect, direct effect, and total effect decomposition.

	*B*	Boot SE	Boot LLCI	Boot ULCI	(%)
Total effect	0.290	0.013	0.265	0.315	
Direct effect	0.203	0.013	0.177	0.230	70.14
The mediating effect of stressful life events	0.087	0.009	0.071	0.104	29.86

### Testing for moderated mediation

**Figure 2:**
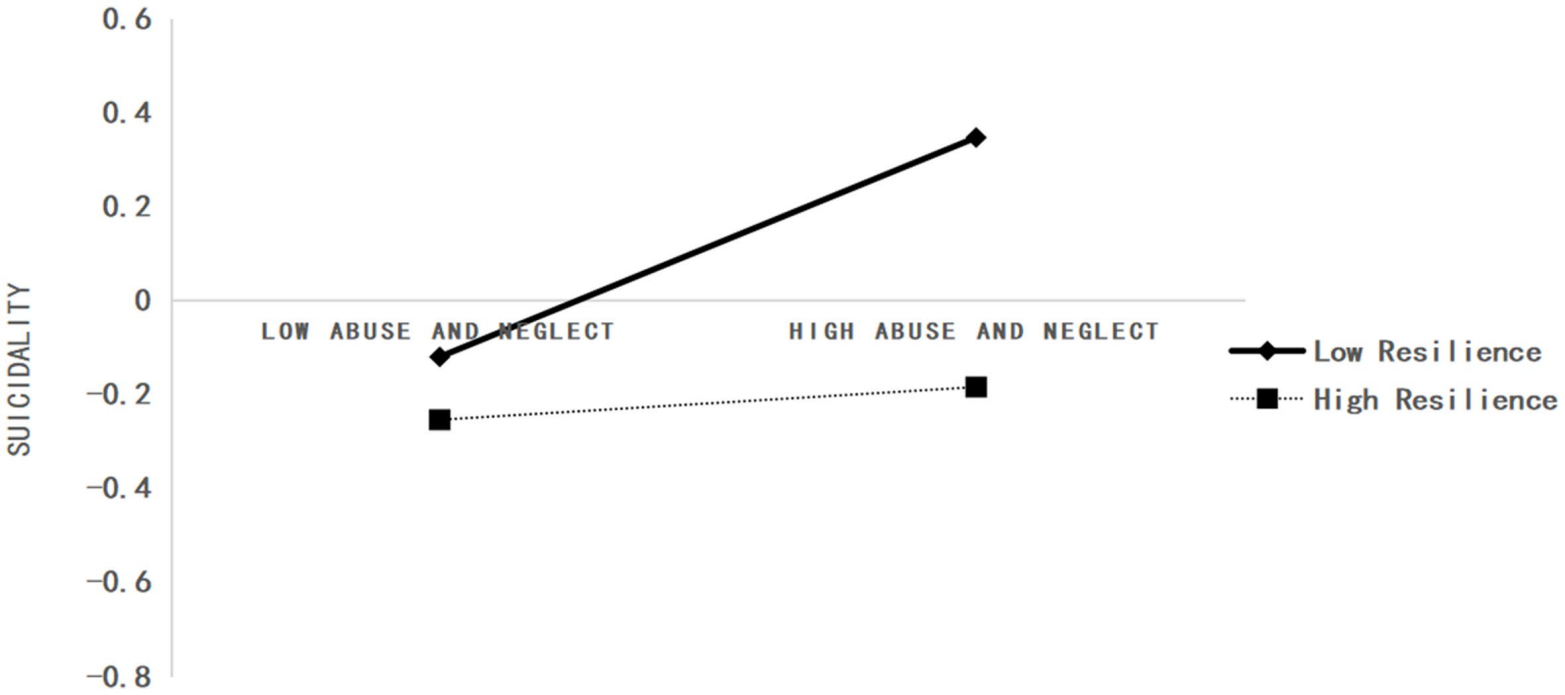
The moderating role of resilience in the relationship between abuse and neglect and suicidal behavior.

**Figure 3:**
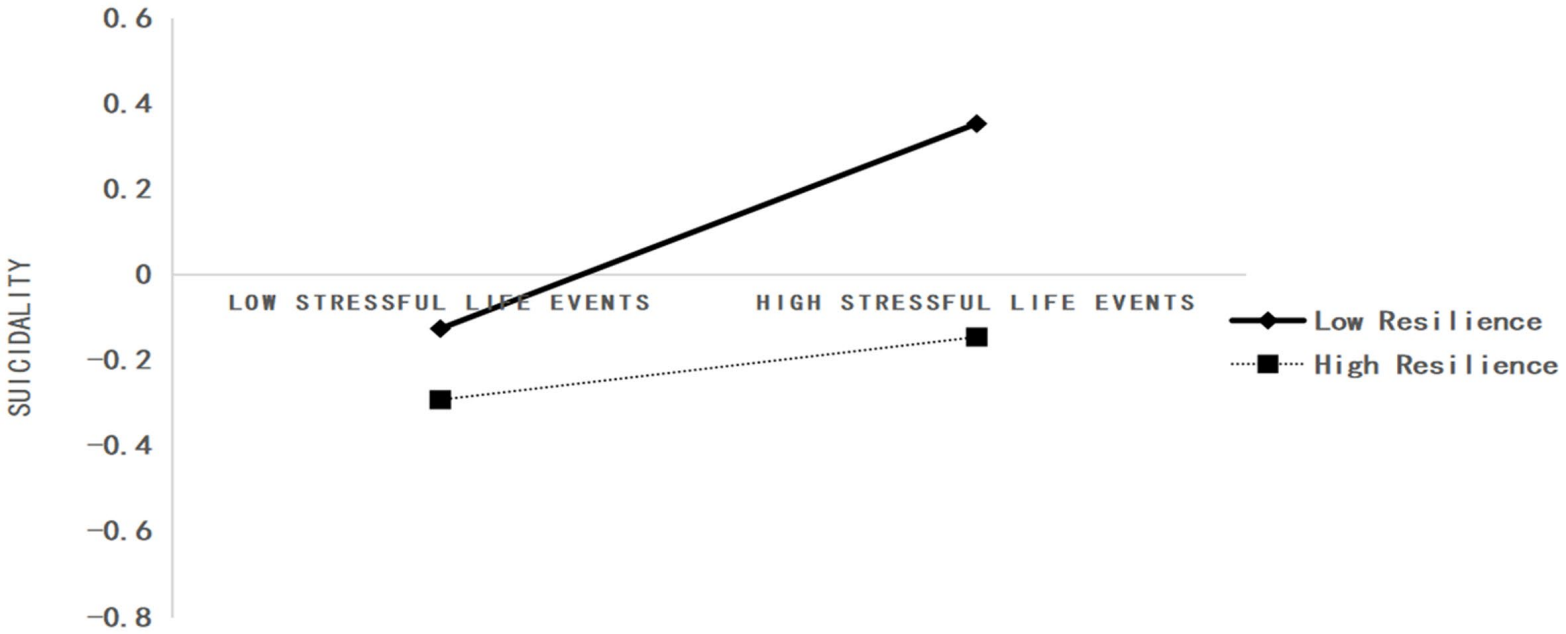
The moderating role of resilience in the relationship between stressful life events and suicidal behaviors.

Table 6 presents the moderating effect test results for resilience. After including resilience into the model, the product of abuse and neglect and resilience (*β* = −0.08, *t* = −6.09, and *p* < 0.001), stressful life events, and resilience (*β* = −0.10, *t* = −7.12, and *p* < 0.001) had a significant predictive effect on suicidality. This revealed that resilience played a moderating role in the relationship between abuse and neglect and stressful life events with suicidality. Further simple slope analysis showed (see Figures 2, 3), that for subjects with a low level of psychological resilience (M − 1SD), as the level of neglect and abuse increased, suicidal behaviors showed a significant increase (simple slope = 0.22, *t* = 13.67, and *p* < 0.001). For subjects with a higher level of resilience (M + 1SD), with the increase in the level of abuse and neglect, suicidal behavior demonstrated a significant increasing trend (simple slope = 0.05, *t* = 2.22, and *p* < 0.05); however, the increase was small. Therefore, the predictive effect of abuse and neglect on suicidal behavior weakened as the level of resilience increased. As Figure 3 displays, for subjects with a low level of psychological resilience (M − 1SD), with an increase in stressful life events, suicidal behavior showed a significant upward trend (simple slope = 0.10, *t* = 7.37, and *p* < 0.05); for those with a higher level of resilience (M + 1SD), with an increase in the level of stressful life events, suicidal behavior showed a significant upward trend (simple slope = 0.02, *t* = 2.17, and *p* < 0.05), but the increase was smaller. Therefore, the predictive effect of stressful life events on suicidal behavior weakened as the level of resilience increased.

**Table 6:** Moderated mediation model testing.

IV	DV			
	Model 1 (DV: Stressful life events)		Model 2 (DV: Suicidality)	
	*B*	*t*	*B*	*t*
Gender	−0.026	−2.137*	0.060	4.965***
Age	0.044	3.717***	0.004	0.324
Abuse and neglect	0.404	33.776***	0.134	9.372***
Stressful life events			0.156	11.257***
Resilience			−0.166	−12.776***
Abuse and neglect × Resilience			−0.083	−6.092***
Stressful life events × Resilience			−0.099	−7.120***
*R*	0.408		0.403	
*R^2^*	0.166		0.163	
*F*	388.537***		162.101***	
Conditional direct effect analysis at values of the resilience (M ± SD)				
	B	SE	LLCI	ULCI
2.90 (M − 1SD)	0.056	0.004	0.046	0.063
3.39 (M)	0.034	0.004	0.027	0.041
3.89 (M + 1SD)	0.013	0.006	0.002	0.025
Conditional indirect effect analysis at values of the resilience (M ± SD)				
	B	Boot SE	Boot LLCI	Boot ULCI
2.90 (M − 1SD)	0.026	0.003	0.020	0.033
3.39 (M)	0.016	0.002	0.013	0.020
3.89 (M + 1SD)	0.006	0.003	0.001	0.012

DV, dependent variable; IV, independent variable.

## Discussion

Our results showed that abuse and neglect could positively and significantly predict adolescent suicide, supporting Hypothesis 1. These results are consistent with previous findings (Duprey et al., 2020). Ignoring childhood abuse can reflect substantial adversity in a critical family environment. Adolescents usually rely on caregivers for safety, protection, and nurturing; abuse and neglect from caregivers can lead to despair, prompting adolescents to build a negative attitude toward the future, which may lead them to committing suicide (Berardelli et al., 2022). Additionally, previous studies have shown that abuse and neglect can destroy the active adaptation ability of individuals in terms of motivation, attitude, emotion, and/or interpersonal relationships. Consequently, adolescents who experienced child abuse and neglect have poor positive adaptive ability and related psychological functions, making it impossible to for them to adequately cope with the negative effects, which leads to an increase in suicide risk (Yates, 2004).

Second, stressful life events are important factors leading to suicide in adolescents; this is consistent with previous research findings (Díaz-Oliván et al., 2021; Cohen et al., 2022). However, we expanded the scope of research and confirmed that stressful life events mediate the relationship between adolescent abuse, neglect, and suicide. This finding suggests that abuse and neglect, as chronic stressors, increase adolescents’ vulnerability and sensitivity to stressful life events and further impair their ability to adapt and cope with stress, thereby increasing suicide risk, supporting Hypothesis 2.

Exposure to adverse environments is directly implicated in the development of psychopathology, aggression, and suicidality (Keene and Epps, 2016; Mclafferty et al., 2018). Anderson and colleagues revealed greater risk for suicide attempts among those reporting exposure to more than one subtype of maltreatment (Anderson et al., 2002). Any factor related to suicide could be a positive or negative confounder for other factors and mediate or moderate the effects on suicidality (Lee et al., 2015). The results showed that stressful life events partially mediated the relationship between abuse and neglect and suicide, that is, abuse and neglect could indirectly affect adolescent suicide through stressful life events. In other words, adolescents who experience childhood abuse and neglect are more likely to be affected by stressful life events that lead to suicide. Furthermore, the combination of stressful life events, abuse, and neglect can have dose–response effects and increase suicide risk among adolescents. According to the self-trauma model (Reece, 2002), complex trauma can lead to the maladaptive development of three main abilities of individual emotion regulation, self-identity and interpersonal relationship. Therefore, when adolescents with abuse and neglect experiences face a new stressful environment, they are unable to rely on and use the above three internal resources; this possibly pushes them to commit suicide or indulge in other risky behaviors.

Additionally, this study’s most important findings illustrate individual differences in the predictive effects of abuse, neglect, and stressful life events on suicidality. Both the direct effects of abuse and neglect on suicidality and indirect effects of stressful life events were moderated by resilience. These effects were stronger for adolescents with lower resilience, supporting Hypothesis 3. These results suggest that resilience, as a positive adaptive capacity, can help mitigate the potential adverse effects of abuse, neglect, and stressful life events in adolescent suicides.

In the face of adversity and stressful life events, a positive response, and attitude are important life adaptabilities; resilience represents this ability. Highly resilient individuals exhibit better skills that help them respond actively to negative environmental impacts, including emotional regulation, problem-solving ability, hopefulness, and support seeking. Everyone has innate resilience, but it will increase or decrease with the change of living environment (Mampane, 2014). Studies have shown that abuse and neglect can cause significant and lasting damage to adolescents’ physical and mental health, reduce their resilience to stressful life events, and increase risk behaviors such as suicide (Wang et al., 2019). In addition, suicide will make individuals more susceptible to stressful life events in their future life, and the occurrence of stressful life events will in turn reduce the resistance and adaptation ability of abusive and neglected individuals to stressful life events, leading teenagers to fall into a vicious circle, further increasing their suicide risk. However, highly resilient adolescents have higher levels of hope, optimism, and problem-solving skills that can help them mobilize internal and external protective resources to buffer against the negative effects of abuse and neglect and stressful life events. At the same time, adolescents with high resilience have a positive outlook on life and believe that adversity (abuse and neglect, stressful life events) is temporary, external and has limited influence, which enhances their ability to resist adversity and reduces the risk of suicide (Horwitz et al., 2017; Chen and Kuo, 2020). Therefore, resilience may buffer the priming effects of abuse, neglect, and stressful life events on suicide.

### Limitations

While these findings provide evidence for increased resilience being a protective factor for suicidal tendencies, but there are still some limitations. First, due to the study was limited by the cross-sectional nature of the data, the data did not allow inference of cause and effect; therefore, cross-sectional surveys and longitudinal studies should be combined in the future to further track the relationship between childhood abuse and neglect, stressful life events, resilience and adolescent suicide. Second, we did not consider the specific content of the variables. Future research should extend our findings by evaluating stressful life events, neglect, and physical abuse in specific domains, such as learning stressors, sexual abuse, and emotional neglect. Third, given that the living conditions of adolescents are closely related to their risk of abuse and neglect, future research should stratify the living conditions of adolescents to identify and control for groups with high rates of abuse and neglect, such as welfare systems and refugees. Fourth, in view of the lasting effects of abuse and neglect on adolescents, long-term follow-up studies should be conducted on adolescents suffering from severe abuse and neglect in the future, in order to further explore the long-term effects of abuse and neglect in the future work, life and study of adolescents. Finally, suicide is a multifactorial disease with multiple interacting predictors, and relationships between these factors may be difficult to detect. Therefore, in the future, more rigorous research design and diversified research methods should be adopted to summarize the most important predictors of adolescent suicide, so as to provide more scientific basis for the prevention of adolescent suicide.

## Conclusions and implications for practice

This study used structural equation model to investigate the impact of abuse and neglect on suicide among Chinese adolescents, and examined the mediating effect of stressful life events and the moderating effect of resilience. The results showed that there was a positive relationship between abuse and neglect and suicide among Chinese adolescents. In addition, stressful life events and resilience significantly influenced adolescent suicide, and mediated and moderated the relationship between abuse and neglect and suicide among Chinese adolescents. These results indicate the importance of suicide and abuse and neglect in the physical and mental health of adolescents.

In addition, the moderated mediation model in this study provides implications for the prevention of adolescent suicide. First, regarding abuse and neglect, schools should regularly screen and identify high-risk groups and establish different levels of prevention and control of early warning systems. Parents should be encouraged to participate in their child’s learning, life, and growth. They should adopt the right parenting style. Second, schools and families should actively focus on the psychological and emotional changes of children at different stages, communicate with children effectively, help them identify stressful life events, and offer correct guidance and solutions. School psychological workers can begin with psychological resilience and formulate corresponding intervention strategies, such as cognitive behavioral therapy, to change irrational thinking and instill hope. Additionally, they should provide psychological resilience training that cover cognitive reconstruction, problem-solving, and positive coping. These improve the ability of adolescents, who experience abuse and neglect and stressful life events, to adapt and cope with adversity, advance their hopes and aspirations for the future, and ultimately reduce their risk of suicide.

## Data availability statement

The original contribution to this study is included in the article and that further inquiries can be directed to the first author, HC (changhj0812@126.com).

## Ethics statement

All procedures performed in studies involving human participants were approved by the Ethics Committee of Xinxiang Medical University (#XYLL-2018015). Written informed consent to participate in this study was provided by the participants or their legal guardians/next of kin.

## Author contributions

HC designed the study. ZY and YZ collected data. HC analyzed the data and wrote the manuscript. PS and JC have revised the manuscript. All authors have contributed to the manuscript and approved the submitted version.

## Funding

This study was supported by the National Natural Science Foundation of China (grant number: 81803252).

## Conflict of interest

The authors declare that the research was conducted in the absence of any commercial or financial relationships that could be construed as a potential conflict of interest.

## Publisher’s note

All claims expressed in this article are solely those of the authors and do not necessarily represent those of their affiliated organizations, or those of the publisher, the editors and the reviewers. Any product that may be evaluated in this article, or claim that may be made by its manufacturer, is not guaranteed or endorsed by the publisher.

## Footnotes

^1^ http://afhayes.com/index.html
